# A rapid method for selecting suitable animal species for studying pathogen interactions with plasma protein ligands *in vivo*

**DOI:** 10.1111/1751-7915.12601

**Published:** 2017-02-07

**Authors:** Clément Naudin, Ariane Schumski, Outi M. H. Salo‐Ahen, Heiko Herwald, Emanuel Smeds

**Affiliations:** ^1^ Department of Clinical Sciences LundDivision of Infection MedicineLund UniversityLundSweden; ^2^ Faculty of Natural Sciences and Engineering, PharmacyÅbo Akademi UniversityTurkuFinland; ^3^ Present address: Institute for Cardiovascular PreventionLudwig‐Maximilians UniversityMunichGermany

## Abstract

Species tropism constitutes a serious problem for developing relevant animal models of infection. Human pathogens can express virulence factors that show specific selectivity to human proteins, while their affinity for orthologs from other species can vary significantly. Suitable animal species must be used to analyse whether virulence factors are potential targets for drug development. We developed an assay that rapidly predicts applicable animal species for studying virulence factors binding plasma proteins. We used two well‐characterized *Staphylococcus aureus* proteins, SSL7 and Efb, to develop an ELISA‐based inhibition assay using plasma from different animal species. The interaction between SSL7 and human C5 and the binding of Efb to human fibrinogen and human C3 was studied. Affinity experiments and Western blot analyses were used to validate the assay. Human, monkey and cat plasma interfered with binding of SSL7 to human C5. Binding of Efb to human fibrinogen was blocked in human, monkey, gerbil and pig plasma, while human, monkey, gerbil, rabbit, cat and guinea pig plasma inhibited the binding of Efb to human C3. These results emphasize the importance of choosing correct animal models, and thus, our approach is a rapid and cost‐effective method that can be used to prevent unnecessary animal experiments.

## Introduction

The total number of proteins in blood plasma is not known, but a fairly recent study using mass spectrometric analyses suggests that human plasma contains around 2000 different proteins (Farrah *et al*., 2011). Bacterial pathogens such as *Staphylococcus aureus* (*S. aureus*) have different cell wall‐anchored or secreted proteins that bind to various human plasma proteins, such as fibrinogen (Fg), complement and antibodies (Serruto *et al*., 2010; Sidorin and Solov'eva, 2011). The interactions with these host proteins may help the pathogen to evade the immune system or cause pathologic reactions. The binding of bacterial proteins to plasma proteins is, however, not ubiquitous to all animal species. The selectivity is highly variable and sometimes the binding appears to be human specific, such as the interaction of *S. aureus* bone sialoprotein binding protein (Bbp) with human fibrinogen (hFg), which is highly specific as Fg from other species tested (cat, dog, cow, sheep, mouse, pig) is not bound by Bbp (Vazquez *et al*., 2011). In contrast, *S. aureus* clumping factor A (ClfA) has a very similar structure as Bbp, but ClfA binds to Fg from several species, including human, but not sheep Fg (Geoghegan *et al*., 2010). To develop new therapies against bacterial pathogens, an attractive approach is to target bacterial virulence factors that help bacteria to evade the immune system or cause pathologic host responses. As a proof of concept, these assays need to be performed in suitable laboratory animals. However, there are currently no robust or simple assays available for choosing the most applicable laboratory animal species. Hence, this study was set out to develop such a method. As it would be too laborious and cost‐intensive to rely upon the purification of the corresponding plasma proteins from various animals, we decided to build a competitive ELISA‐based method where the binding of bacterial virulence factors to purified human plasma proteins is studied in the presence of plasma from different animal species.

The two different *S. aureus* proteins that were chosen for this approach are staphylococcal superantigen‐like protein 7 (SSL7) and extracellular fibrinogen binding protein (Efb). SSL7 is a secreted protein that binds to IgA and complement C5 (Laursen *et al*., 2010), thereby inhibiting IgA binding to its Fc receptor. This interaction helps the pathogen in immune evasion (Langley *et al*., 2005). Efb is a small protein that has high affinity for complement C3 (Lee *et al*., 2004b) and fibrinogen (Bodén and Flock, 1994). It has been proposed that Efb binds to Fg and C3 simultaneously to protect the pathogen from phagocytosis (Ko *et al*., 2013).

This study was undertaken to develop a simple ELISA‐based method to study the interaction of bacterial virulence factors to host plasma proteins from different animal species. To see whether we could corroborate our findings structurally, we also developed a model of the Efb‐C3 complex for two species whose C3 was not bound by Efb and compared those models with the experimentally determined complex of Efb bound to human C3 (Hammel *et al*., 2007).

## Results

### Plasma inhibition assay of SSL7 binding to human C5

**Figure 1:**
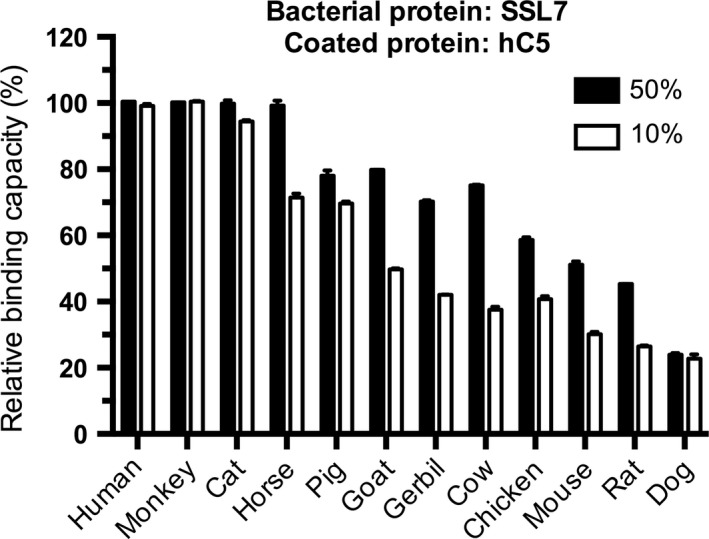
Animal plasma inhibition of SSL7 binding to coated human complement C5. Citrated plasma from animals was pre‐incubated with SSL7 followed by addition to microtiter plates coated with human complement C5 (1 μg per well; 5 pmol). Bound SSL7 was detected by rabbit polyclonal anti‐TEV followed by anti‐rabbit‐HRP conjugate. After washing, the absorbance was measured at 450 nm. The assay was performed in triplicate, and the results are shown as mean ± SD. The relative binding capacity (reBC) was calculated as described in Experimental Procedures (Appendix S1), and species were ordered according to decreasing reBC using 10% plasma. To make the plasma inhibition of SSL7 binding to coated human C5 easier to follow, the inhibition was inversed to a graph displaying relative binding capacity (reBC) as detailed in Experimental Procedures (Appendix S1) and also put in order of decreasing reBC at 10% plasma.

To establish rapid and cheap assays that do not require purification of target plasma proteins from various animal species, we decided to focus on a competitive ELISA‐based approach. To this end, the human protein of interest is immobilized onto an ELISA plate followed by an incubation step with the respective bacterial virulence factor resuspended in plasma from different animal species. Binding of the bacterial protein is then monitored by specific antibodies (Fig. 1).

One suitable bacterial virulence factor to study is SSL7, as this protein has been studied in the context of binding to plasma proteins from a number of different species (Langley *et al*., 2005) and there is also structural information available for this protein (Laursen *et al*., 2010). First, purified human complement C5 was coated on microtiter plates and SSL7 was incubated with it in varying concentrations to yield a concentration‐dependent graph (data not shown). From this graph, a concentration of SSL7 that falls into the linear range was chosen. In the next series of experiments, the selected concentration of SSL7 was pre‐incubated in tubes with diluted citrated plasma (CP) samples (10% and 50%) from different species (including human). The mixtures were then added to microtiter plates precoated with human C5 and the bound SSL7 was measured as described in *Experimental Procedures* (Appendix S1). As shown in Fig. 1, human plasma had high relative binding capacity (reBC). The results further demonstrated that different species varied significantly in their reBC. As the main goal of the method was to identify suitable species for testing the bacterial protein binding, one option was to set the bar for the binding species fairly high. In this study, this bar was set to species where the reBC ≥ 80% when using 10% plasma. Using this approach, we classified three species, that is human, monkey and cat, as strong binders. The remaining nine species were further categorized as intermediate binders (horse, pig, goat, gerbil, chicken) with a reBC in the range of 40% to 80% and non‐binders with a reBC < 40% (cow, mouse, rat, dog) in 10% plasma.

### Plasma inhibition assay of Efb binding to human fibrinogen and C3

**Figure 2:**
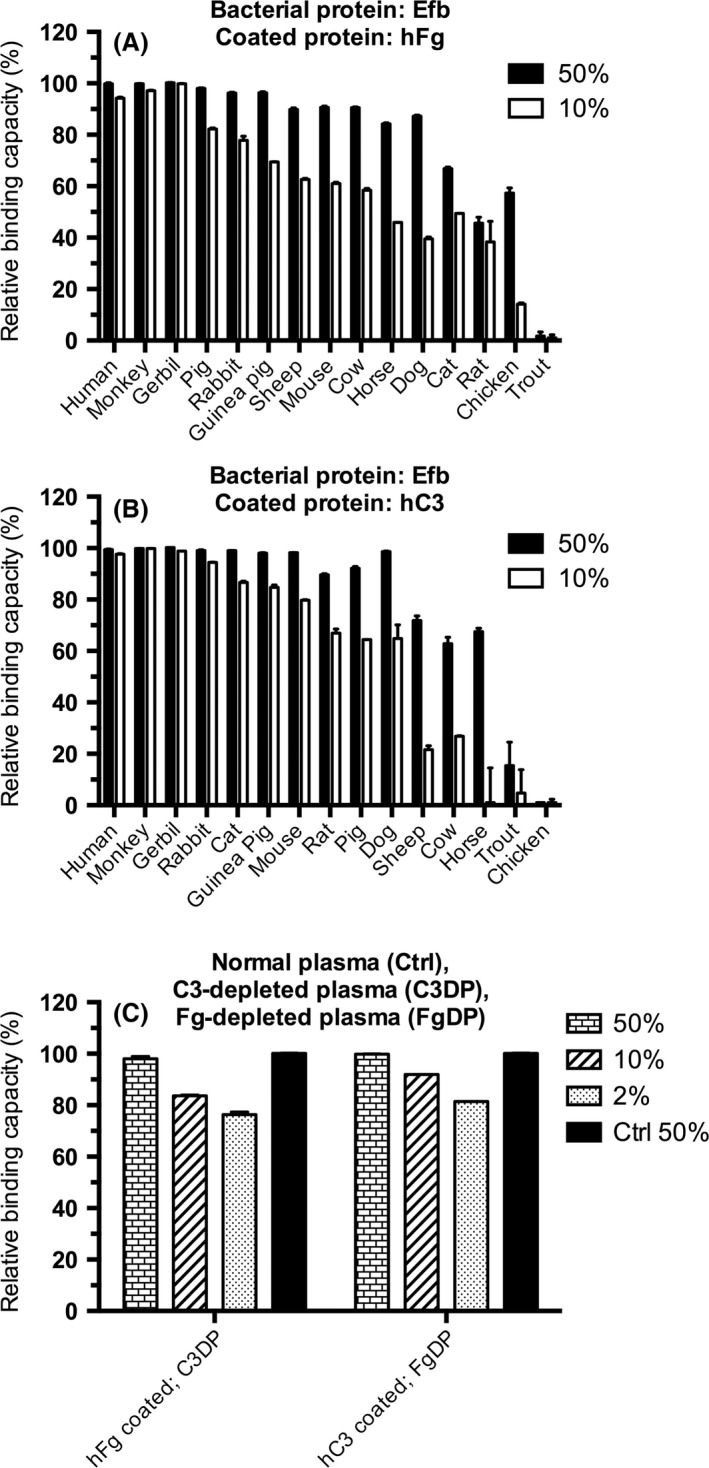
Animal plasma inhibition of Efb binding to coated human fibrinogen and human C3. Citrated plasma from animals was pre‐incubated with Efb followed by addition to microtiter plates coated with human fibrinogen (hFg; A) or human C3 (hC3; B). Citrated human plasma depleted of either C3 (C3DP) or Fg (FgDP) or normal plasma (Ctrl) was pre‐incubated with Efb and added to microtiter plates coated with hFg or hC3 (C). Bound Efb was detected by anti‐His HRP conjugate and absorbance measured at 450 nm. The assay was performed in triplicate, and the results are shown as mean ± SD. The relative binding capacity was calculated as described in Experimental Procedures (Appendix S1), and species were ordered according to decreasing reBC using 10% plasma.

To test that the method is not restricted to SSL7, we next employed Efb (Bodén and Flock, 1994; Lee *et al*., 2004b) and tested its binding to human fibrinogen and C3 in the presence of plasma from different animal species. As shown in Fig. 2A, when microtiter plates were coated with human Fg, some animal CP displayed a high reBC, whereas others showed a lower reBC. The animal species with the binding activity similar to human CP were monkey, gerbil and pig; these can be considered as strong binders (reBC ≥ 80% at 10% plasma). On the other hand, species such as rat and chicken displayed a very low binding. Together with trout, the only fish species in the study, these species were defined as non‐binders. Some species displayed an intermediate binding in the assay (rabbit, guinea pig, sheep, mouse, cow, cat, horse and dog). Interestingly, rat did not appear to be a good choice for *in vivo* testing related to the Fg‐binding of Efb.

The same inhibition approach was also used when the interaction of Efb with human C3 was analysed. The assay generated somewhat different results, where several species were found to be strong binders (human, monkey, gerbil, rabbit, cat, guinea pig, mouse), while three species were defined as intermediate binders (rat, pig and dog) and five species as non‐binders (sheep, cow, horse, trout and chicken; Fig. 2B). Overall, the binding pattern had some differences in regard to the binding to Fg and C3. As Efb has two known ligands (Fg and C3), there was a risk that binding of one of the ligands could interfere with binding of the other ligand. To exclude this, we performed assays where we used citrated human plasma depleted of either C3 or fibrinogen. As demonstrated in Fig. 2C, Efb bound to human depleted plasma for both proteins, C3 or Fg, at a similar level to normal CP (Ctrl) with a high reBC as expected (reBC > 80% at 10% plasma), suggesting that binding of Efb to coated Fg was independent of plasma C3 concentration. Similarly, the binding of Efb to coated C3 was independent of plasma Fg concentration.

### Identification of plasma ligands from different animal species by agarose bead affinity assays

**Figure 3:**
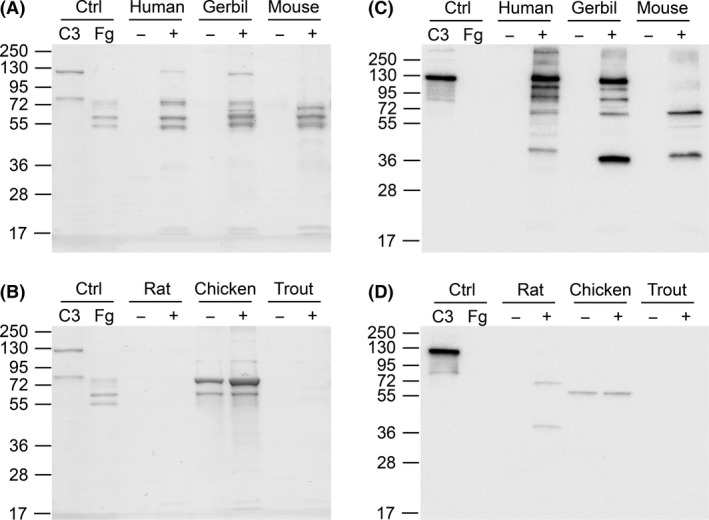
Plasma pull‐down experiment using agarose beads binding to Efb. Human citrated plasma was added to agarose beads in presence (+) or absence (−) of Efb. Upon washing of the beads, eluted samples were analysed using 10% SDS‐PAGE under reducing conditions and stained with Coomassie (A, B). Samples were also analysed under reducing conditions using Western blot analysis with an anti‐human C3d antibody followed by anti‐rabbit‐HRP conjugate (C, D). As a control, purified hC3 (1 μg) and hFg (1 μg) were loaded.

As the ELISA inhibition assays can be used to predict suitable species even in a system with two different plasma ligands, we wished to corroborate these results using another already established method. To this end, we employed a method similar as previously described by Langley *et al*. (2005). A few representative human Fg and C3 binder and non‐binder species were selected. Agarose beads conjugated with anti‐His antibodies were incubated with CP from different species pre‐incubated with His‐tagged Efb. Upon washing of the beads, proteins bound to the beads were eluted. As Fg and C3 migrated differently in the gel, the two ligands are easily discriminated. Whereas human and gerbil C3 bound to Efb, C3 was absent in the eluates from mouse, rat and trout CP. In contrast, human, gerbil and mouse Fg bound Efb, whereas rat and trout Fg did not (Fig. 3A,B). Chicken could not be properly analysed in regard to Fg as the plasma sample clotted both in the presence and absence of Efb (Fig. 3A,B).

To further corroborate these results, Western blot analysis was used to detect the C3d domain of different species as Efb binds to human C3d (Lee *et al*., 2004a). Intact C3d that was in complex with Efb was detected with both human and gerbil plasma, whereas the other species did not show a signal for intact C3d (Fig. 3C, D). However, when testing mouse and rat plasma, intermediate bands were detected (also present in human and gerbil profiles), which may suggest that these species are binders as defined by the ELISA inhibition assay (Fig. 2B).

### Protein modelling of chicken and trout complement C3 corroborates loss of binding to Efb

**Figure 4:**
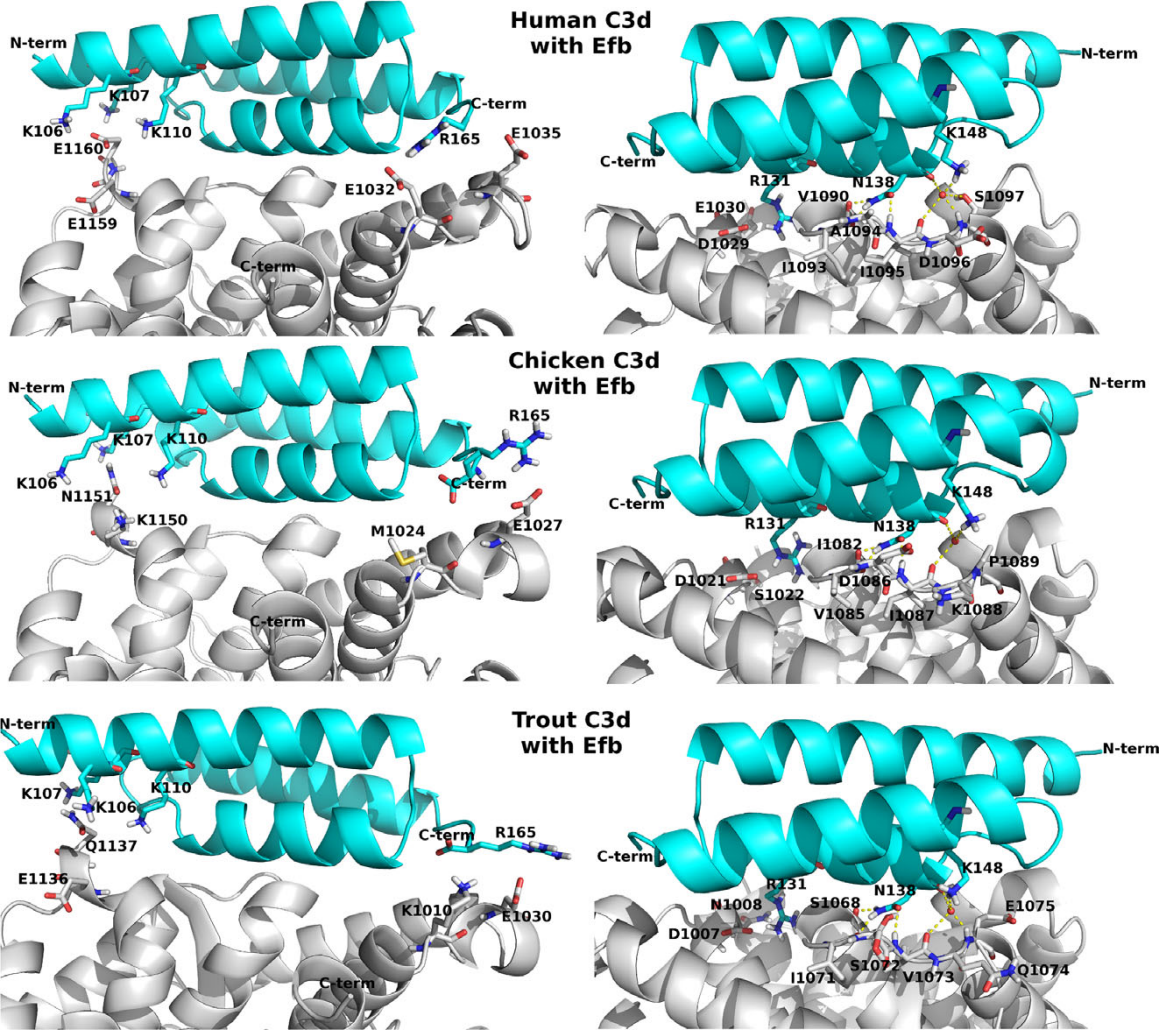
Key interface interactions between the complement component C3d (light grey cartoon representation) and the *S. aureus* Efb (cyan cartoon representation). Top panel – human C3d (PDB ID: 2GOX); middle panel – chicken C3d model; bottom panel – trout C3d model. The right side figures are showing the same protein complex as on the left side after rotating the complex 180 degrees about the origin. Corresponding key residues in each interacting protein are shown as sticks; non‐polar hydrogen atoms are omitted for clarity. Atom colour code: carbon – grey/cyan; oxygen – red; nitrogen – blue; sulfur – yellow; hydrogen – white. Hydrogen‐bonding interactions are denoted as yellow dashed lines. A conserved interface water molecule that is part of the hydrogen‐bonding network at the interface is represented as a red sphere.

To investigate the molecular reason for the lack of Efb binding to chicken and trout complement C3, we constructed comparative models of the chicken and trout C3d domains using the Efb‐C–human C3d crystal structure as a template. We also compared the molecular electrostatic potentials (MEPs) of the human, chicken and trout C3d structures. The binding interaction between the positively charged Efb and the negatively charged human C3d has been characterized to be driven by the electrostatic complementarity of the two proteins (Hammel *et al*., 2007; Haspel *et al*., 2008). Most Efb residues at the interface are engaged in hydrogen‐bonding or electrostatic interactions. A detailed computational study of the pairwise interactions in the human C3d‐Efb complex suggests that the interface residues that contribute the most to the binding free energy all build networks of salt bridges (Haspel *et al*., 2008). The chicken and trout C3d structural models reveal significant differences among the key interface residues compared with the human C3d (Table 1; Fig. 4).

**Table 1:** Key interface residues of the human C3d‐Efb complex and the corresponding residues in the chicken and trout C3d models

Interacting residues		Corresponding residues in the C3d models	
Efb‐C	Human C3d	Chicken C3d	Trout C3d
R131	D1029, E1030	D1021, **S1022** a	D1007, **N1008**
R131	N1091	N1083	**T1069**
N138	I1093, A1094, I1095	V1085, **D1086**, I1087	I1071, **S1072**, V1073
K135	D1156	**K1147**	**S1133**
K145	D1096	**K1088**	**Q1074**
K148	I1095, D1096, S1097	I1087, **K1088**,** P1089**	V1073, **Q1074**,** E1075**
D156	K1050	K1042	**N1028**
K106, K107, K110	E1159, E1160	**K1150**,** N1151**	E1136, **Q1137**
R165	E1032, E1035	**M1024**, E1027	**K1010**, E1013

**a.** Residues denoted in bold differ significantly from the corresponding human residue by their physicochemical properties.

For example, several negatively charged residues in the human C3d are instead positively charged or neutral residues in the chicken C3d model. Moreover, in the chicken C3d there is a proline residue (P1089) in the corresponding position to S1097 of human C3d. In the human C3d‐Efb complex this serine forms two hydrogen bonds to a conserved interface water that mediates the interactions with the Efb residues R148 and N138. These hydrogen bonds are not possible for a proline (Fig. 4, right side top and middle). On the other hand, the corresponding trout C3d residue E1075 can form only one hydrogen bond with a water molecule. Most of the positively charged residues of the human C3d interface are neutral (polar) or in one case is of opposite charge (E1032 human; K1010 trout) in the trout C3d model (Table 1). Moreover, the trout model lacks a hydrogen bond from the side‐chain of T1069 to R131, an interaction that is present in both the human C3d‐Efb crystal and the chicken C3d‐Efb model as they possess an asparagine in that position.

**Figure 5:**
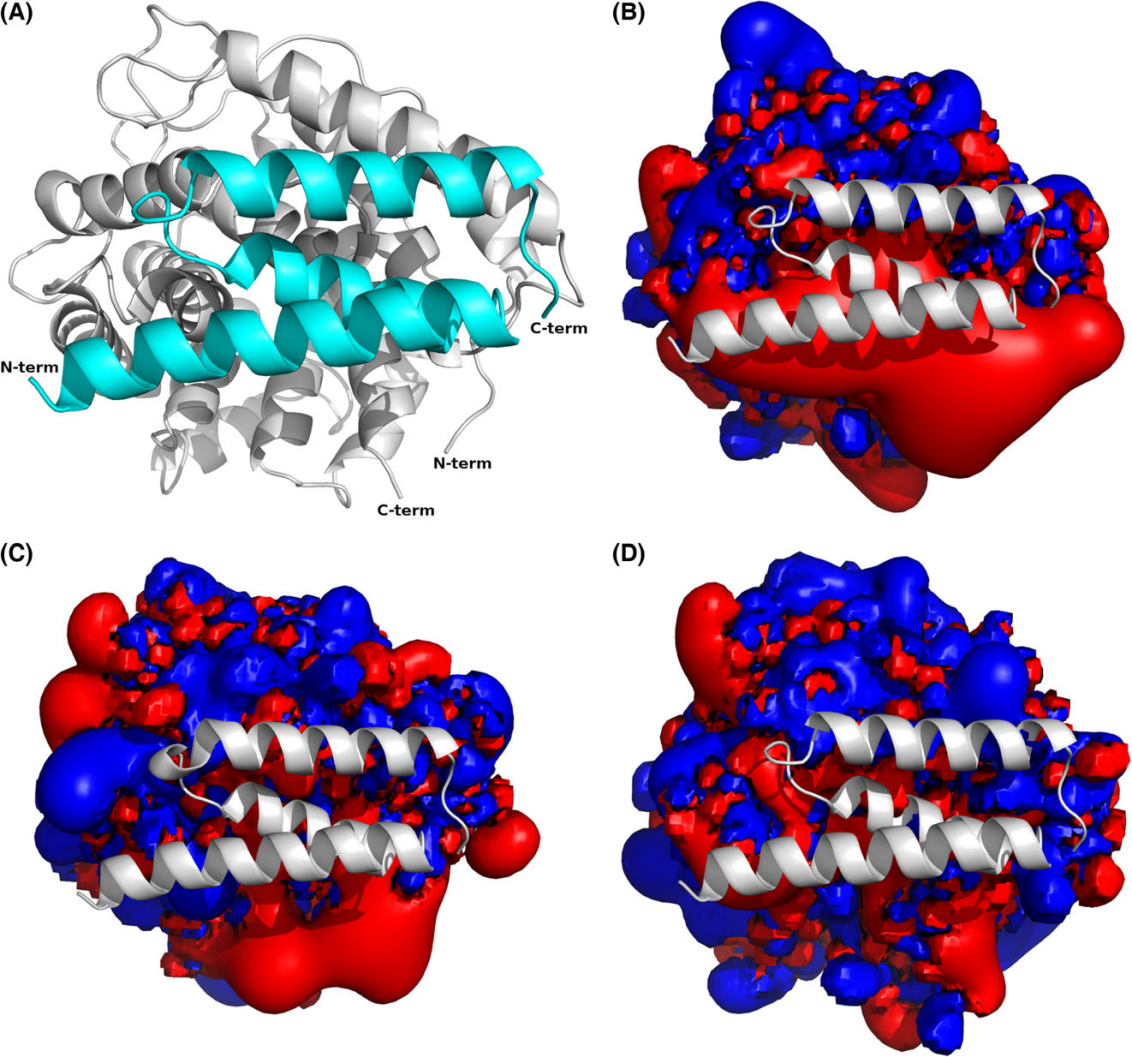
Efb binding interface of the complement component C3. A. Human C3d (light grey) bound to *S. aureus* Efb protein (cyan; PDB ID: 2GOX); cartoon presentation of the proteins. Molecular electrostatic potentials of the human (B), chicken (C) and trout (D) C3d domain at the Efb‐C3 binding interface. The potential maps are visualized at the energy level −1/+1 kT (negative potential in red and positive potential in blue). The orientation of the proteins is the same as in (A). Efb in its binding site is shown in white cartoon representation.

Haspel *et al*. (2008) reported that the Efb‐C double‐mutant R131A/N138A lost the affinity for human C3d and the respective single mutants exhibited a reduced affinity for this complement component. These residues are among the key residues that contribute the most to the binding energy. If only one or two key residue mutations are enough to disrupt the complex formation, it is an expected result that the chicken C3d cannot bind to Efb. These residue differences at the chicken and trout C3d interface disrupt important salt bridges and hydrogen‐bonding networks that are present at the binding interface of the human C3d‐Efb complex. The molecular electrostatic potential maps of the C3d interface indeed look different for all the three C3d proteins (Fig. 5).

### Prediction of suitable animal species

In this report, it has been demonstrated that it is possible to use unfractionated CP from different animal species to determine the species selectivity of bacterial proteins. The best available information in regard to species tropism from the literature is available for SSL7, where several animal species have been described as binders (rabbit, goat, sheep, pig and primates human, chimpanzee and baboon), while several others (cow, sheep, mouse) are listed as non‐binders in regard to their affinity to C5 (Langley *et al*., 2005). Our analyses are in line with the published results as seen from Table 2.

**Table 2:** Relative binding capacity of animal plasma in regards to the binding of SSL7 to hC5 and binding of Efb to hFg and hC3

	SSL7 binding to C5		Efb binding to Fg		Efb binding to C3	
	This study	Literature	This study	Literature	This study	Literature
Human (Positive control)	++	++^a^	++	++^b^	++	++^c^
Chimpanzee	ND^d^	++^a^	ND^d^	NR^e^	ND^d^	NR^e^
Baboon	ND^d^	++^a^	ND^d^	NR^e^	ND^d^	NR^e^
Cynomolgus monkey	++	NR^e^	++	NR^e^	++	NR^e^
Cow	−	−a	+	NR^e^	−	NR^e^
Sheep	ND^d^	Unclear a	+	NR^e^	−	NR^e^
Pig	+	Unclear a	++	NR^e^	+	NR^e^
Mouse	−	−a	+	NR^e^	++	NR^e^
Rabbit	ND^d^	++^a^	+	NR^e^	++	NR^e^
Rat	−	−a	−	NR^e^	+	NR^e^
Goat	+	Unclear a	ND	NR^e^	ND^d^	NR^e^
Horse	+	−a	+	NR^e^	−	NR^e^
Chicken	+	NR^e^	−	NR^e^	−	NR^e^
Dog	−	NR^e^	+	NR^e^	+	NR^e^
Cat	++	NR^e^	+	NR^e^	++	NR^e^
Gerbil	+	NR^e^	++	NR^e^	++	NR^e^
Guinea pig	ND^d^	NR^e^	+	NR^e^	++	NR^e^
Trout	ND^d^	NR^e^	−	NR^e^	−	NR^e^

The relative binding capacity is defined as in Experimental Procedures (Appendix S1). The binding is defined as: strong binder (++), intermediate binder (+) and non‐binder (−) according to the criteria in the Results section. For the literature, the different species are only grouped as strong binder (++), unclear binder (Unclear) or non‐binder (−).

**a.** The data on binding of SSL7 to C5 from different species are from (Langley *et al*., 2005).

**b.** Data from Bodén and Flock (1994).

**c.** Data from Lee *et al*. (2004b).

**d.** ND = not determined or inconclusive result due to antibody issues.

**e.** NR = no results known in the scientific literature.

*Staphylococcus aureus* can cause a range of infections in various animals and it is therefore interesting that the species selectivity for various plasma proteins are very different for the many *S. aureus* virulence factors. Broadly, if using the human plasma ligand as the reference point, there are three different categories: restricted specificity (binding to only one of the tested species, e.g. Bbp binding only to human Fg; Vazquez *et al*., 2011), intermediate specificity (at least two binders and non‐binders each, e.g. SSL7 binding to some species but not to others; Langley *et al*., 2005) and broad specificity (maximum one of the tested species is a non‐binder, e.g. ClfA binds to several animal species Fg, but not to sheep Fg; Geoghegan *et al*., 2010). It is therefore important to categorize the Efb binding to the two plasma ligands into these categories. For both Fg and C3, the Efb specificity would best be categorized as broad. Interestingly, there are important differences in the binding to these two ligands however.

A particular challenge in interpretation of these kinds of inhibition experiments using CP relates to the fact that many bacterial proteins bind to several host ligands and this is also the case with the proteins studied in this report, i.e. Efb (Fg, C3) and SSL7 (IgA, C5). It can therefore be speculated that the presence of other ligands in plasma can result in inhibition and falsely be interpreted as a binding species. There are, however, structural biology data that display that SSL7 can bind IgA and C5 simultaneously (Laursen *et al*., 2010). Also in the case of Efb, investigators have proposed simultaneous binding of Fg and C3 (Ko *et al*., 2013). Our results with C3‐ and Fg‐depleted human plasma showed that the assay worked well whether only one or both ligands were present in the plasma.

In this report, we have demonstrated the usefulness of a simple assay to determine which laboratory animal species that have plasma ligands binding to a bacterial protein and hence suited for testing *in vivo*. If this method is put into general use among investigators studying bacterial proteins, a number of unnecessary and potentially also painful animal experiments can easily be avoided.

Furthermore, we can conclude that in the absence of experimental protein–protein complex structures, bioinformatics and computer‐aided comparative protein modelling can be used for studying the molecular level interactions between the host and pathogen proteins. Such models can corroborate the experimental data in explaining why the plasma proteins of certain species bind the bacterial proteins and why those from others do not. Before experimental assays, these models can also be used for predicting if certain proteins will bind to each other or not. Further, the knowledge of the molecular level protein–protein interactions can be utilized in the design of new therapies targeting bacterial proteins.

## Conflict of Interest

ES and CN have filed a patent application in regard to the method described herein. ES is co‐inventor on a US patent and a US patent application regarding interactions of bacterial proteins with fibrinogen.

## Supporting information


**Appendix S1.** Experimental procedures.
