# Supplementary material for: A rapid method for selecting suitable animal species for studying pathogen interactions with plasma protein ligands in vivo
Source: Microb Biotechnol. 2017 Feb 7;10(3):657–65. doi: 10.1111/1751-7915.12601 (PMC5404189; doi:10.1111/1751-7915.12601)
Supplement: Supplementary file 1 — Appendix S1. Experimental procedures. [file MBT2-10-657-s001.doc]

**Supplemental Information**

**Experimental Procedures**

**Bacterial protein expression**

Plasmids coding for two different constructs of SSL7 were a kind gift from Dr Gregers Rom Andersen, Aarhus University, Aarhus, Denmark. SSL7 was expressed as a fusion protein containing both a 6xHis tag and a TEV tag using the modified pET32-Ek/LIC plasmid with TEV site (Novagen). For SSL-7 expression and purification, competent *E. coli* BL21 (DE3) was transformed by heat shock with SSL-7 plasmid and colonies containing plasmid selected using LB agar plates with 100 µg/ml ampicillin.

*Escherichia coli* M15 (pREP4) (Thermo Fisher Scientific) (Waltham, MA, USA) (pQE) (Qiagen) containing plasmids encoding N-terminal 6xHis-tagged Efb fusion proteins were grown overnight at 37 °C in LB containing 100 µg/ml ampicillin and 25 µg/ml kanamycin (kind gift from Dr Christine Heilmann, University Hospital of Münster, Germany). The overnight cultures were diluted 1:60 into fresh LB medium, and recombinant protein expression was induced with 1 mM isopropyl β-D-1-thiogalactopyranoside (IPTG) for 4 h. Bacteria were harvested by centrifugation and lysed using lysing buffer (PBS, 10 mM imidazole, pH 7.4, 1 mg/ml Lysozyme, Protease Inhibitor tablet EDTA free, 10 µg/ml DNase, 5 mM MgCl_2_) followed by sonication (6 x 10s bursts, 5s break, 150 watts, Model S-150D, Branson Ultrasonics, Danbury, CT, USA). After centrifugation, supernatants were filtered (0.22 µm) and soluble proteins purified through Ni^2+^ chelating chromatography (GE Healthcare, Uppsala, Sweden). Purified proteins were concentrated and buffer exchanged into PBS and stored at -20 °C. Protein concentrations were determined by NanoDrop (model ND-1000, Thermo Fisher Scientific).

**Blood plasma and antibodies**

Human citrated plasma was purchased from Seralab Ltd (West Sussex, UK) or taken from human volunteers, whereas animal plasma was purchased from Seralab. C3-deficient plasma was from Assaypro (St. Charles, MO) and Fg-deficient plasma from Affinity Biologicals (Ancaster, ON, Canada). Human C3 was from HyCult (Plymouth Meeting, PA), human Fg from Sigma (St Louis, MO), and human C5 from Calbiochem (Solna, Sweden). Rabbit polyclonal anti-His antibodies directly conjugated with HRP were purchased from Abcam (Cambridge, UK) and rabbit polyclonal anti TEV cleavage site antibodies were purchased from Thermo Fisher Scientific (Waltham, MA, USA).

**ELISA inhibition assays.**

Microtiter plates (F96 MaxiSorp Nunc-Immuno plate, Thermo Fisher Scientific, Roskilde, Denmark) were coated overnight at 4 °C with 1 µg protein (hC5, hC3 or hFg). During the blocking step of the wells with 2% BSA in PBS, citrated plasma at 50% (v/v) and 10% (v/v) was incubated separately with 2.5 pmol bacterial protein (SSL-7 or Efb) in PBS, 2% BSA for 30 min at room temperature (RT). Samples were then added to the wells and plates incubated for 1 h at RT. After washing with PBS-T, horseradish peroxidase (HRP)-conjugated anti-His antibodies (1:10000) or anti-TEV site antibodies (1:1000) were added for 1h at RT. To detect binding of anti-TEV site antibodies plates were washed and a HRP-Anti-Rabbit antibodies (1:3000, Sigma) were added for 1 h. Bound antibodies conjugated with HRP were detected by using 3,3’,5,5’-Tetramethylbenzidine (TMB) (Thermo Fisher Scientific, Waltham, MA) and the presence of bacterial protein quantified by measuring the resulting absorbance at 450 nm in an ELISA microplate reader (Victor3, PerkinElmer, Waltham, MA) and analyzed using the Excel software.

**Protein modeling of Efb binding to complement C3**

Comparative models of chicken and trout complement C3d domains were built using the Modeller v. 9.15 software (Sali and Blundell, 1993) (Webb and Sali, 2014). The template structure was obtained from the Protein Data Bank (PDB; [www.rcsb.org](http://www.rcsb.org/); (Berman et al., 2000)): the human C3d complexed with *S. aureus* Efb-C (PDB ID: 2GOX; resolution 2.2 Å; (Hammel et al., 2007)). The amino acid sequences of the complement component C3 for human, chicken, trout and seven other organisms were obtained from the UniProt Knowledgebase (UniProt, 2015); human (P01024), chicken (*Gallus gallus*, Q90633), trout (*Oncorhynchus mykiss*, P98093), bovine (*Bos taurus*, Q2UVX4), pig (*Sus scrofa*, P01025) mouse (*Mus musculus*, P01027), rat (*Rattus norvegicus*, P01026), guinea pig (*Cavia porcellus*, P12387), rabbit (*Oryctolagus cuniculus*, P12247), Thai catfish (*Clarias macrocephalus*, F5HT30). The multiple sequence alignment of the C3 sequences was carried out with Clustal Omega (<http://www.ebi.ac.uk/Tools/msa/clustalo/>; (Sievers et al., 2011)) and the PSIPRED 3.3 server (<http://bioinf.cs.ucl.ac.uk/psipred/>; (Buchan et al., 2013)) was used to predict the secondary structures of the chicken and trout C3d domains. The alignment was manually adjusted to avoid gaps in the predicted secondary structure regions. Six crystal water molecules residing at the human C3d-Efb interface were included in both the models. See Supplementary material for the MODELLER input alignments.

Molecular modeling software package Maestro (versions 9.3.518 and 10.3.015) (Schrödinger, LLC) was used to refine the chicken and trout C3d-Efb models and the human C3d-Efb crystal complex: hydrogen atoms were added at pH 7.0, missing side chains were added to the crystal structure, histidine protonation states were manually adjusted. The crystal water was kept in the C3d-Efb structures while adding the hydrogen atoms and optimizing the hydrogen bond network. A restrained energy minimization of hydrogen atoms was performed to all the structures using the OPLS 2005 force field (Banks et al., 2005) and the heavy atom positions were further optimized in the model structures only (convergence to RMSD 0.3 Å compared to the original positions). The UHBD program (Madura et al., 1995) was used to calculate the molecular electrostatic potentials (MEPs) of the proteins. The protein models and MEPs were visualized with PyMOL (Schrödinger, LLC).

**Agarose bead affinity assays**

Efb (3 µM) was incubated with agarose beads conjugated with mouse anti-His antibodies (Sigma) in PBS, 2% BSA, at 4 °C overnight. Agarose beads (25 µl) treated with Efb were washed with PBS followed by addition of 50% plasma in PBS, 2% BSA in a final volume of 1 ml for 1h in RT under gentle agitation. As a control, beads incubated in buffer instead of Efb were used. After washing, beads were resuspended in 100 µl glycine buffer, pH 2 for 10 min at RT, centrifuged at 5000 x g for 5 min and the supernatant mixed with 0.1M Tris-HCl, pH 8.5 and 5x sample buffer. Samples were heated at 95 °C for 5 min and then analyzed under reducing conditions on 10% SDS-PAGE. Purified proteins (hC3 and hFg) were loaded on gel as control. In a Western blot experiment, after electrophoresis, proteins were transferred to a polyvinyl membrane and incubated with polyclonal rabbit anti-hC3d antibodies (DAKO, Glostrup, Denmark). After addition of an HRP-conjugated secondary antibody, immunostained proteins were detected using a chemiluminescence kit (SuperSignal West Pico Chemiluminescent Substrate, Thermo Scientific, Waltham, MA) and analysis on a ChemiDoc system (Bio-Rad).

**Calculation of relative binding capacity**

The assay is based upon an inhibition assay where a reduction in signal actually indicates a binding of the bacterial protein to one or more plasma ligands from the animal plasma being tested. Hence, the inhibition is converted into a term called relative binding capacity (reBC). Complete binding of bacterial protein to plasma from a tested animal was set to 100% reBC. The relative binding capacity (**reBC**) in percent is then defined as:

*reBC* =
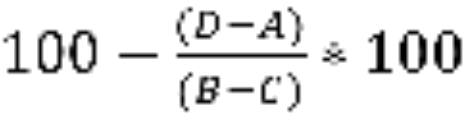


where

***A*** = absorbance with only plasma present, but no bacterial protein. Microtiter plate coated with the purified plasma ligand.

***B*** = absorbance without plasma, but in presence of bacterial protein. Microtiter plate coated with the purified plasma ligand.

***C*** = absorbance without plasma, but in presence of bacterial protein. Microtiter plate has no coated ligand.

***D*** = absorbance upon premixing of the bacterial protein with plasma from a certain species at a desired plasma concentration (e.g. mouse plasma 50%). Microtiter plate has the coated ligand.

Typically several different plasma concentrations from different animal species are used in the assay. Upon premixing of the bacterial protein with different dilutions of plasma from a certain species (e.g. 10% or 50% mouse plasma) an absorbance value ***D*** is read in the spectrophotometer. ***ReBC*** can then be defined for different species and different concentrations of plasma, for example 50% mouse plasma. Note: in our assays shown in the figures, the value ***C*** was so low (always even lower than ***A***) that it could be disregarded in the calculations.
